# The Ammonia Adsorption and Desorption Behavior of Nafion

**DOI:** 10.3390/membranes15050149

**Published:** 2025-05-14

**Authors:** Dominik Sachse, Andreas Glüsen, Klaus Wippermann, Martin Müller, Uwe Rau, Ralf Peters

**Affiliations:** 1 Institute of Energy Technologies (IET-4)—Electrochemical Process Engineering, Forschungszentrum Jülich GmbH, 52428 Jülich, Germany; a.gluesen@fz-juelich.de (A.G.);; 2 Jülich Aachen Research Alliance (JARA-Energy), Faculty of Electrical Engineering and Information Technology, RWTH Aachen University, Schinkelstr. 2, 52062 Aachen, Germany; 3 Institute of Energy Materials and Devices (IMD-3)—Photovoltaics, Forschungszentrum Jülich GmbH, 52428 Jülich, Germany; 4 Faculty of Mechanical Engineering, Ruhr-Universität Bochum, Synthetic Fuels, Universitätsstr. 150, 44801 Bochum, Germany

**Keywords:** electrochemical nitrogen reduction reaction, Nafion, membranes, ammonia, contamination

## Abstract

The electrochemical nitrogen reduction reaction (eNRR) for electrochemical ammonia (NH_3_) synthesis is considered a promising alternative to the energy-intensive and highly CO_2_-emitting Haber-Bosch process. In numerous experiments, the Nafion membrane has been used as an electrolyte or separator. However, Nafion adsorbs and desorbs NH_3_, leading to erroneous measurements and making reproducibility extremely difficult. This study systematically investigates the interaction between NH_3_ and Nafion, underscoring the strength of the interaction between ammonium-ions (NH_4_^+^) and protons (H^+^). We found that minute quantities of synthesized NH_3_ are prone to persist within the membrane, albeit without affecting the ion conductivity and resistivity of Nafion. Consequently, the removal of NH_3_ from the membrane can occur under conditions where synthesis is not viable. The objective of this work is to heighten awareness regarding the interaction between NH_3_ and Nafion and contribute to the attainment of reliable and reproducible outcomes in eNRRs.

## 1. Introduction

Ammonia (NH_3_) is one of the most widely produced chemicals in the world, with an annual production exceeding 170 million tons [1,2]. NH_3_ and compounds derived from it play a crucial role in the agricultural, pharmaceutical, and textile industries. Notably, more than 80% of the NH_3_ produced is utilized for the production of fertilizers [3,4]. Without the industrial production of NH_3_ the present world population would not exist and could not be fed [5,6].

In addition to these factors, NH_3_ has been gaining increasing attention in the energy sector due to its potential as an energy carrier. This interest stems from its high H_2_ content of 17.8 wt%, and the energy density of 15.6 MJ L^−1^ of liquefied NH_3_, which is 70% higher than that of liquid H_2_, 9.1 MJ L^−1^ at a cryogenic temperature, making NH_3_ viable as a carbon-neutral energy carrier [7]. Additionally, the transport and storage of NH_3_ is simple compared to H_2_, as it can be liquefied and transported at 20 °C and an excess pressure of 8.6 bar [4]. Furthermore, it can be employed as a fuel in a direct ammonia fuel cell or indirectly in a hydrogen fuel cell [8]. It can also utilized as a fuel in gas turbines or generators [9].

However, for more than a hundred years, NH_3_ has been synthesized using the Haber-Bosch process. However, for more than a hundred years, NH_3_ has been synthesized using the Haber-Bosch process. This process is extremely energy intensive, consuming about 1% of the power generated globally [10] and is responsible for 1 to 2% of annual CO_2_ emissions [11,12,13]. The production of 1 t results in the generation of approximately 2 t of CO_2_ [14]. Given the substantial energy consumption and significant CO_2_ emissions entailed by the Haber-Bosch process, there is a compelling need to enhance the efficiency, sustainability, environmental-friendliness, and reduce the CO_2_ emissions of NH_3_ synthesis [12]. Furthermore, the decentralization of NH_3_ production is important for reducing dependencies in individual production countries and lowering transport and associated energy costs. Hence, the electrochemical NH_3_ synthesis is considered a promising, environmentally-friendly method for converting N_2_ into NH_3_ in mild conditions (below 100 °C) by means of adsorption, activation and desorption processes [15]. A CO_2_-neutral production process can be achieved if renewable energy is used for electricity supply and the utilization of air and water as reactants.

However, ammonia production rates are still fairly low and are hardly confirmed by the contribution of the electrochemical nitrogen reduction reaction (eNRR). This is due to the fact that different non-negligible contaminants affect the synthesis and result in false amounts of NH_3_ being detected. There are several sources of contamination [16,17]. One major issue is the absorption of NH_3_ in the electrolyte. In numerous cases, the proton-conducting membrane, Nafion, is used as the electrolyte and separator between the anode and cathode [18,19,20,21]. Due to the basic character of NH_3_, it reacts with the protons of Nafion, forming NH_4_^+^ ions, i.e., the NH_4_^+^-form of Nafion. This in turn has a strong influence on the unequivocal determination of the NH_3_ production rate, as atmospheric NH_3_ pollution, impurities in the feed-gas stream like NH_3_ itself, and impurities present within the electrochemical cell can persist in the form of NH_4_^+^ within the membrane, thereby complicating the analysis of NH_3_ produced by eNRR. The NH_4_^+^-ions resulting from the impurities can be expelled from the membrane and subsequently dissociate into NH_3_ at the cathode, leading to a falsely interpreted production rate of NH_3_ particularly with respect to the catalyst and operating parameters. Conversely, if NH_3_ is produced within the cell, it may be adsorbed by the membrane, thereby preventing the detection of NH_3_ at the outlet of the cell. This outcome leads to a false negative result. Moreover, the NH_4_^+^-ions within the membrane have been demonstrated to significantly affect the ion conductivity and ohmic resistance of the electrolysis system [22,23,24,25,26,27].

The objective of this study is to enhance comprehension of the adsorption behavior of NH_3_/NH_4_^+^ in Nafion. The investigation aims to determine the specific conditions and extent to which the NH_4_^+^-ions introduced into the membrane are expelled as NH_3_ from the cell especially from the membrane. In addition, this study considers the influence of the desorbed NH_4_^+^ on the properties of the Nafion membrane. The paper commences with an in-depth analysis of the adsorption and desorption behavior exclusively within the Nafion membrane. Subsequently, Nafion is integrated into an electrochemical cell configuration and its behavior meticulously examined under the dynamic influence of electrochemical reactions.

## 2. Materials and Methods

### 2.1. Nafion

**Figure 1:**
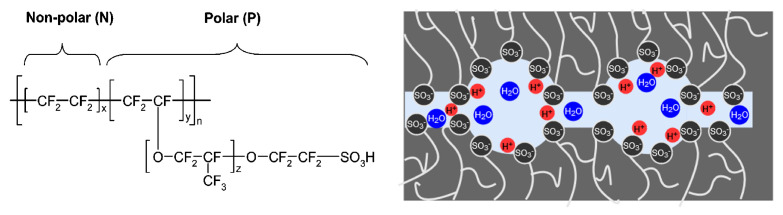
Nafion structural formula, adapted from ref. [28] and a schematic illustration of the microstructure of Nafion.

Nafion^®^ from Chemours^™^ (Tyrone, Wilmington, DE, USA) is a perfluorinated membrane, whose ion conductivity is attributable to the presence of the ionic sulfonic acid groups (R-SO_2_-OH). Figure 1 displays the structural formula [28] and a schematic illustration of the channel-shaped microstructure of Nafion. It is utilized in an acidic or neutral environment due to its good proton conductivity, chemical, and mechanical stability. Based on its excellent properties, Nafion is the standard material used as a proton-conducting membrane and as a separator in electrolysis processes under these environmental conditions [29].

In this study, Nafion 115, with a thickness of 127 μm and size of 42 * 42 mm^2^, was used. The average weight was determined, based on five dried membrane pieces, yielding an average of 443.2 mg with an error of ± 15.3 mg. The pieces were dried in an oven at 80 °C for 1 h before the weight was measured.

The equivalent weight is defined as the value of the amount of substance of the sulfonic acid groups (-SO_3_H) per gram of dry polymer. For Nafion, this value is 1100 g mol^−1^ [30]. With respect to the weight of the membrane pieces, these contain 403 μmol ± 14 μmol of sulfonic acid groups.

### 2.2. Ion Exchange

**Figure 2:**
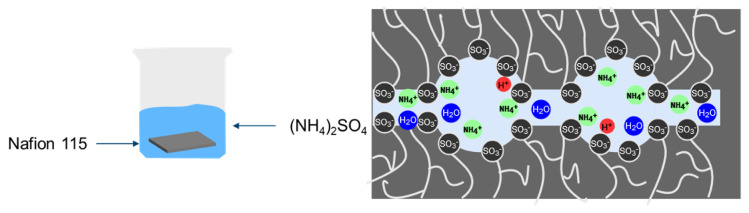
An Illustration of the ion exchange process and the NH_4_^+^-form of Nafion.

To introduce NH_3_ in the form of NH_4_^+^-ions into the membrane, the protons (H^+^), which are bound to the sulfonic acid groups, must be exchanged with NH_4_^+^-ions. This exchange occurs in a one-to-one ratio. Thus, the maximum amount of NH_3_/${\mathrm{NH}}_{4}^{+}$, that can be bound inside the polymer structure is 403 μmol ± 14 μmol. Ammonium sulfate ((NH_4_)_2_SO_4_) is utilized as the NH_4_^+^ source. Figure 2 depicts the principle of the ion exchange. Nafion 115 is introduced into a 25 mL (NH_4_)_2_SO_4_ solution. The immersion time was varied in order to investigate its influence. The ion exchanged Nafion membrane in NH_4_^+^-form is also depicted in Figure 2.

The study aims to investigate the influence of the quantity of ${{\mathrm{NH}}_{4}}^{+}$ in the solution and on the ion exchange. For this reason, (NH_4_)_2_SO_4_ solutions were prepared with different quantities of substance (n) of ${{\mathrm{NH}}_{4}}^{+}$-ions in relation to the amount of $H^{+}$ in the membrane. The ratio 1:1 (Table 1) therefore means that the amount of ${{\mathrm{NH}}_{4}}^{+}$ in the solution is the same as the amount of $H^{+}$ in the membrane. To gain the specific amount of substance of NH_4_^+^, the mass (*m*) of (NH_4_)_2_SO_4_ must be calculated using the following equation (Equation (1)):

(1) $$m\left({\left({\mathrm{NH}}_{4}\right)}_{2}{\mathrm{SO}}_{4}\right)=\frac{\beta ({{\mathrm{NH}}_{4}}^{+})\;\cdot \;V\;\cdot \;M({\left({\mathrm{NH}}_{4}\right)}_{2}{\mathrm{SO}}_{4})}{2\;\cdot \;M({{\mathrm{NH}}_{4}}^{+})}$$

where β is the mass concentration inside the volume (*V*) of 25 mL. The molar masses are *M*((NH_4_)_2_SO_4_) = 132.14 g mol^−1^ and *M*(NH_4_^+^) = 18.04 g mol^−1^.

The corresponding quantity of ${{\mathrm{NH}}_{4}}^{+}$ to the ratio, the concentration of ${{\mathrm{NH}}_{4}}^{+}$ (c), the mass concentration of ${{\mathrm{NH}}_{4}}^{+}$ in the prepared (NH_4_)_2_SO_4_ solution, and the required mass of (NH_4_)_2_SO_4_ for preparing the specific ${{\mathrm{NH}}_{4}}^{+}$ concentration are also shown in Table 1.

After the ion exchange took place, the Nafion membrane was dried and introduced into a sulfuric acid (H_2_SO_4_) solution to expel the ${\mathrm{NH}}_{4}^{+}$-ions from the membrane. The H_2_SO_4_ solution facilitates a re-exchange of NH_4_^+^ with H^+^. Subsequently, the amount that was exchanged is analyzed.

The concentration of the H_2_SO_4_ solution is 0.45 mol L^−1^ and the volume is 25 mL, and so a substantial excess of H^+^ is available.

### 2.3. Ammonia/Ammonium Determination

The quantity of NH_4_^+^ in the residual (NH_4_)_2_SO_4_ solution and in the H_2_SO_4_ solution was quantified using the indophenol blue method, which is a common technique for determining NH_3_ levels [4]. This method is particularly adept at measuring especially low concentrations of NH_3_/NH_4_^+^. The indophenol method is based on the method reported by Li et al. and Zhao et al. [31,32]. 2 mL of the sample solution was introduced into a sample vessel. 2 mL of a 1 M sodium hydroxide (NaOH) solution, including sodium salicylate and potassium sodium tartrate was added. The solution contains 5 g of sodium salicylate and 5 g of potassium sodium tartrate in 100 mL of 1 M NaOH. Subsequently, 0.2 mL of a nitroprusside solution was added, containing 0.2 g of nitroprusside that had been dissolved in 20 mL of deionized water. In the end, 1 mL of a sodium hypochlorite solution containing 0.005% sodium hypochlorite was added to the solution. Between each addition, the solution was thoroughly mixed using a vortex mixer. The samples were stored for 30min and subsequently analyzed using the VIS-spectrometer “PV4 Spectrophotometer”, from VWR^™^ at a wavelength of 655 nm in accordance with the methodology proposed by Zhao et al. [31]. In order to achieve suitable concentrations, the samples were either diluted by 1:20 or 1:50.

## 3. Electrochemical Cell

**Figure 3:**
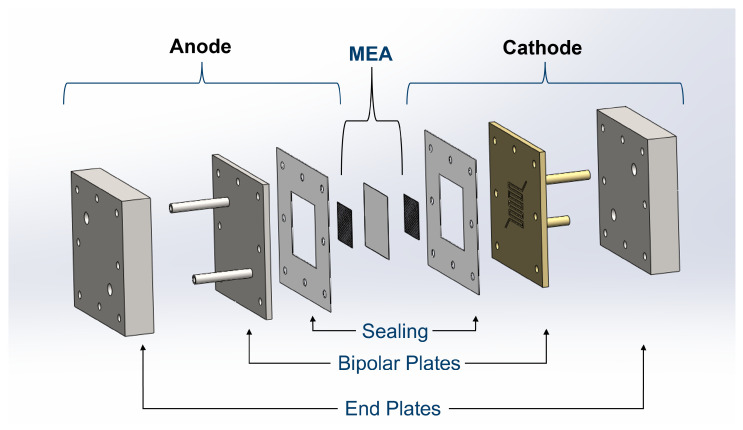
Design of the electrochemical MEA cell and their compounds.

For studying the NH_3_ adsorption and desorption behavior of Nafion 115 in an electrochemical cell, the membrane was assembled into a membrane electrode assembly (MEA). The MEA is the core of an electrochemical cell and is placed in the center of the cell. Figure 3 illustrates the cell set-up. Nafion 115 was introduced into solutions with different concentrations of (NH_4_)_2_SO_4_. 2 mg cm^−2^ of IrO_x_ catalyst, supplied by Alfa Aesar/Thermo Fisher Scientific^™^ (Haverhill, MA, USA) was spray-coated onto a titanium felt substrate from Bekeart^™^, Bekipor^®^ (Zwevegem, Belgium) 2GLD10-0.35. Prior to the spray-coating of the catalyst, a thin Ir-layer was sputter-deposited onto the substrate. This electrode functions as the anode. Knife-coating was employed to deposit 60% Pt/C HiSpec 9100 from Johnson Matthey^™^ (London, UK) onto a carbon felt substrate from Freudenberg (Weinheim, Germany) H2315 CX312. This electrode functions as the cathode. The loading of the catalyst was 1.3– 1.4 mg cm^−2^. Both electrodes were produced in-house. These electrodes are common material for the reactions of the water electrolysis, H_2_O oxidation and H^+^ reduction [33,34]. In order to avoid the transfer of NH_4_^+^ from the membrane to the electrodes and to facilitate disassembling for analysis, a hot-pressing step was not employed. The active cell area was 6.25 cm^2^. To investigate the electrochemical change in more detail, the impedance spectra were fitted with the Zview^®^4 software from Scribner.

The anode and cathode side had identical components installed from the outside to the inside. The end plates were stainless steel and connected to the potentiostat “Octostat 500” from Ivium^®^ (Eindhoven, The Netherlands) which was utilized in all of the experiments and has a maximum current supply of 5 A. The anode bipolar plate was stainless steel coated with platinum, and the cathode bipolar plate was stainless steel coated with gold. The bipolar plates featured a meandering flow field structure. The cell was sealed using PTFE layers, which were customized according to the thickness of the porous transport layer (PTL). The cell assembly process initially involved tightening at a torque of 3 N m, followed by a secondary tightening step at 5 N m. The 5 N m tightening was then repeated to ensure secure sealing.

**Figure 4:**
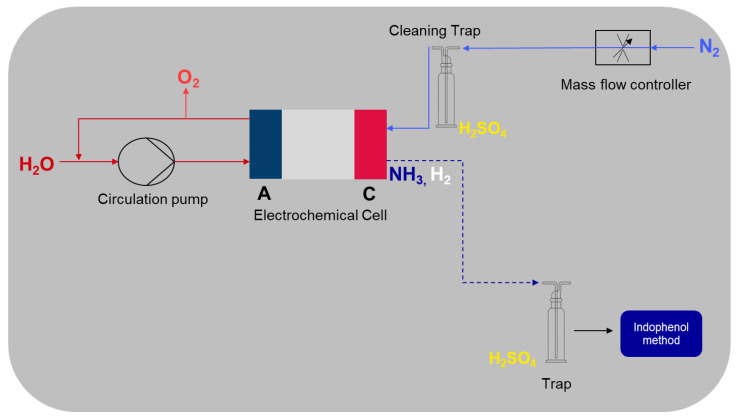
Schematic illustration of the test bench. H_2_O is supplied and O_2_ is produced at the anode. NH_3_ is expelled from the membrane and H_2_ is produced at the cathode. The coloring of the chemicals is done after the CPK color model.

As illustrated in Figure 4, the test rig is composed of several components. Equation (2) describes the oxidation of H_2_O at the anode, through the application of electrical energy, resulting in the production of O_2_. If the resulting H^+^ interacts with NH_4_^+^ within the membrane, NH_4_^+^ is displaced with H^+^ within the membrane. Consequently, NH_4_^+^ desorbs from the membrane. At the cathode, NH_4_^+^ deprotonates, producing NH_3_. The dissociated H^+^ is reduced to H_2_ with the supplied e^−^ from the cathode (Equation (3)).

(2)Anode:        2 H2O⟶4 H++4 e−+O2(3)Cathode: 4 NH4++4 e−⟶4NH3+2 H2

H_2_O was pumped into the anode of the electrochemical cell with a circulation pump. The flow rate of the pump was set at 10 mL/min. On the cathode side, N_2_ was introduced as a carrier gas via a mass flow controller from Brooks Instruments^™^ (Dresden, Germany) to remove NH_3_ from the cell. The cleaning trap filled with H_2_SO_4_ is needed to remove impurities of NH_3_ from the N_2_ feed gas stream, otherwise these impurities can be detected and lead to false results. The trap collects the discharged NH_3_ for subsequent determination via the indophenol method. In the case that H^+^ does not displace NH_4_^+^, only H_2_ is produced in the cathode (Equations (4) and (5)).

(4)Anode:       2 H2O⟶4 H++4 e−+O2(5)Cathode: 4 H++4 e−⟶2 H2

## 4. Results and Discussion

### 4.1. Ion Exchange

#### 4.1.1. Ammonia Adsorption

The thickness and size of the membrane was determined before and after insertion of the membrane pieces in the ammonium sulfate solution using a caliper gauge. Swelling, and an increase in the size and thickness of the membrane, was not detected. The swelling behavior of Nafion in the literature is also different [35,36].

The ion exchange of H^+^ with NH_4_^+^ was studied at temperatures ranging from 25 °C to 80 °C and for different exchange times of 30 and 60 min. The objective was to study the effects of elevated temperatures and extended exchange times. However, it has not been proven that the quantity of the ion exchange increases at higher temperatures. Figure S1, which can be found in the Supplementary Information, illustrates this. It is assumed that the temperature difference is insufficient to influence the equilibrium. In addition, no difference in the quantity of ion exchange was observed when varying the exchange time while the membranes were placed in the same solution concentration at the same temperature (see Figure S2).

**Figure 5:**
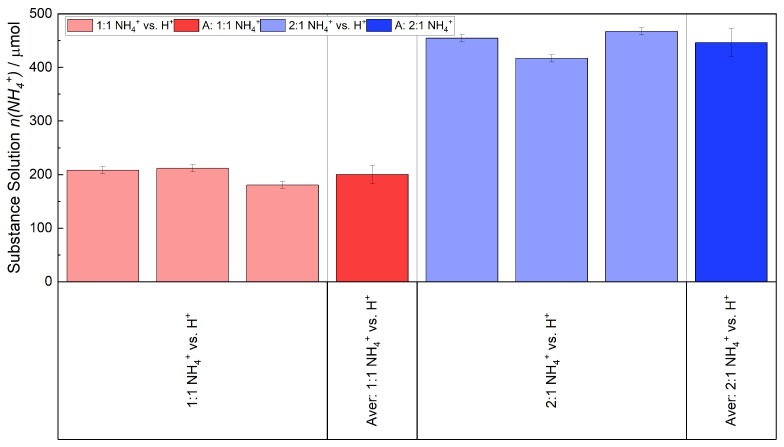
Deviation of the ion exchange for two different ratios of NH_4_^+^ vs. H^+^ (bright colors). The y-axis displays the quantity of NH_4_^+^ remaining in the solution. The average of both proportion is indicated by the red and blue bar.

Despite this, a scattering of results was observed when the experiments were repeated. Figure 5 shows the amount of NH_4_^+^ remaining in the (NH_4_)_2_SO_4_ solution with the respective mean values and error bars for the ratio of ${\mathrm{NH}}_{4}^{+}$ vs. H^+^ 1:1 (red) and 2:1 (blue) (c(NH_4_^+^) = 0.016 and 0.032 mol L^−1^). The ion exchange was conducted at 25 °C (ambient temperature) for a period of 1 h for both ratios. The spread of the average value of the ratio 1:1 was ±17 μmol, and in the case of 2:1, it was ±26 μmol.

The question arises as to whether there is a correlation between the provided quantity of NH_4_^+^-ions and the ion exchange. Table 1 exhibits the diverse proportion of NH_4_^+^ vs. H^+^, which were prepared to subsequently analyze the ion exchange. The ion exchange process of the NH_4_^+^-ions and the H^+^ is an equilibrium-controlled process (Equation (6)). In the initial stage, the NH_4_^+^-ions are exclusively present in the solution (c(NH_4_^+^, Sol)), whereas the protons are located within the membrane (c(H^+^, Mem)). Over time, the process of ion exchange occurs, resulting in the exchange of H^+^ within the membrane with NH_4_^+^. In doing so, the H^+^ desorbs into the solution.

(6) $$c({{\mathrm{NH}}_{4}}^{+},Sol)+c(H^{+},Mem)<=>c({{\mathrm{NH}}_{4}}^{+},Mem)+c(H^{+},Sol)$$

The equilibrium constant (*K_eq_*) is determined through the concentrations of the ions in the media (membrane and solution) after reaching the equilibrium state.

(7) $$K_{eq}=\frac{c({{\mathrm{NH}}_{4}}^{+},Mem)\;\cdot \;c(H^{+},Sol)}{c({{\mathrm{NH}}_{4}}^{+},Sol)\;\cdot \;c(H^{+},Mem)}$$

In light of the assumption that the volume of the solution and the membrane remain constant, it is reasonable to calculate the equilibrium constant using the amount of substance in place of the concentration. As previously outlined in Section 2.2, the quantity of protons present within the membrane is 403 μmol. The quantity of ${{\mathrm{NH}}_{4}}^{+}$-ions in the solution depends on the chosen ratio of ${{\mathrm{NH}}_{4}}^{+}$ against $H^{+}$, as illustrated in Table 1 (${{\mathrm{NH}}_{4}}^{+}$ vs. $H^{+}$). The adsorbed ${{\mathrm{NH}}_{4}}^{+}$-ions within the membrane are quantified through the indophenol method after extracting it from the membrane with an excess of sulfuric acid solution (see Section 2.3). Given that the exchange of ${{\mathrm{NH}}_{4}}^{+}$-ions and $H^{+}$ occurs in one-to-one ratio, the original ${{\mathrm{NH}}_{4}}^{+}$ solution now contains an equivalent quantity of $H^{+}$ to that of ${{\mathrm{NH}}_{4}}^{+}$ within the membrane.

**Figure 6:**
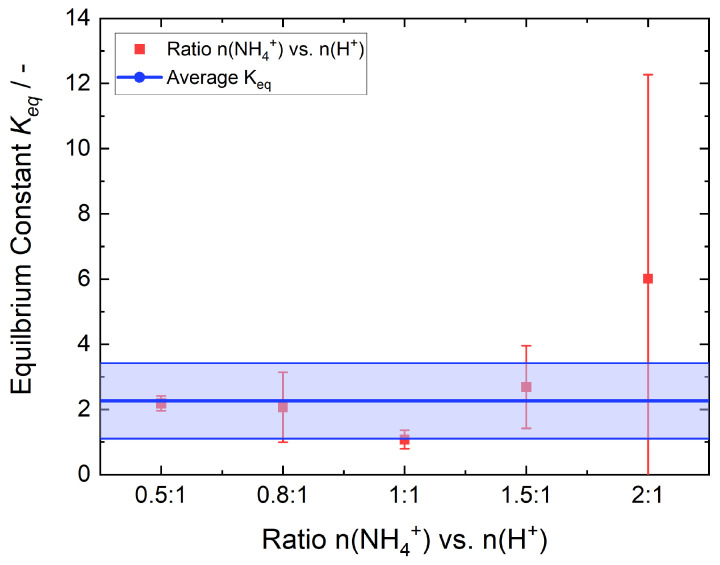
Equilibrium constants of the different ratios of NH_4_^+^ vs. H^+^ (red squares) and their corresponding error bars. The average value of *K_eq_* is shown as the blue line. The colored area is the error of the average value. The value of the ratio 2:1 is not considered for the average value.

Figure 6 shows the determined equilibrium constant of the tested NH_4_^+^ vs. H^+^ ratios as red squares with their respective error bars. As *K_eq_* > 1, the equilibrium is on the product side. Consequently, the ${{\mathrm{NH}}_{4}}^{+}$-ions from the solution prefer to exchange with the $H^{+}$ within the membrane. The error bars of the ratios 0.8:1 and 1.5:1 are larger in comparison to those of 0.5:1 and 1:1, as the determined amount of ${{\mathrm{NH}}_{4}}^{+}$ in the solutions of these ratios showed a larger scatter compared to the latter one. This scattering has a magnifying effect on the error bar of K_*eq*_. Additionally, the error bars of the ratio of 2:1 is very large, because the concentration of $H^{+}$ in the membrane after exchange is calculated by subtracting the concentration of the ${{\mathrm{NH}}_{4}}^{+}$-ions in the membrane after exchange from the concentration of $H^{+}$ in the membrane before the exchange. For the 2:1 ratio, most protons are exchanged and therefore the difference is small and on the order of the magnitude of errors of those of the minuend and subtrahend. This leads to a substantial error in *K_eq_* for the 2:1 ratio. The average value of *K_eq_* is 2.81 ± 2.62, including the ratio of 2:1. If this ratio is not considered for this reason, *K_eq_* is 2.00 ± 1.31, as depicted by the blue line in Figure 6.

#### 4.1.2. Ammonia Desorption

The ion-exchanged Nafion membranes were immersed in a H_2_SO_4_ solution for evaluating the desorption kinetic of NH_3_/${{\mathrm{NH}}_{4}}^{+}$ in relation to time and temperature. This study aimed to elucidate whether increased temperature correlates with increased NH_3_ desorption. No correlation was found. For the desorbed quantity of NH_3_ at room temperature up to 80 °C was similar. Furthermore, we sought to determine the impact of the immersion duration in the H_2_SO_4_ solution on desorption behavior. The desorption time ranged from 10 to 60min. Similar results were obtained. The Nafion pieces used, as noted previously, had a size of $17.64\;{cm}^{2}$ and each one contained 403 μmol ± 14 μmol of $H^{+}$, which were bonded to sulfonic acid groups.

**Figure 7:**
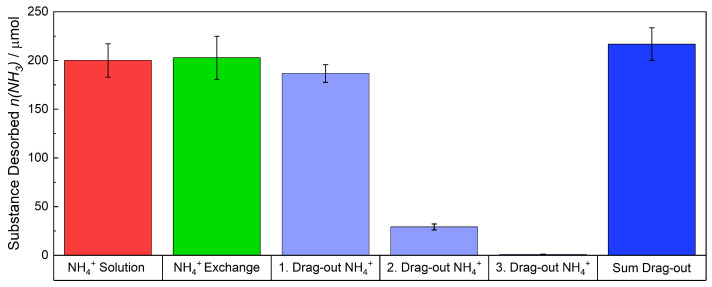
The detected and measured quantities of NH_3_/${{\mathrm{NH}}_{4}}^{+}$ are presented. In red the quantity of ${{\mathrm{NH}}_{4}}^{+}$ that remained in the solution is represented and in green the initial quantity of ${{\mathrm{NH}}_{4}}^{+}$ that was exchanged into the membrane. The blue bar presents the sum of NH_3_ that was driven out from the membrane. In total the membrane was placed three times in a fresh H_2_SO_4_ solution, because an equilibrium of NH_3_ is established between the membrane and the solution, thereby preventing further desorption of NH_3_. These quantities are referred as first to third drag-out, depicted as the bright blue bars.

The red bar in Figure 7, which is the same as in Figure 5, depicts the average remaining quantity of ${{\mathrm{NH}}_{4}}^{+}$ in the solution. 200 μmol of ${{\mathrm{NH}}_{4}}^{+}$ remaining in the solution. The green bar illustrates the amount of ${{\mathrm{NH}}_{4}}^{+}$ that was ion-exchanged into the membrane by subtracting the red bar from the membrane’s initial concentration of $H^{+}$ (403–200 μmol). The ion-exchanged membranes were placed in three fresh H_2_SO_4_ solutions, because during experiments it was found that the ${{\mathrm{NH}}_{4}}^{+}$-ions were not fully removed from the membrane at first. An equilibrium was established between the membrane and the H_2_SO_4_ solution. By replacing the H_2_SO_4_ solution with a fresh one, the desorption of NH_3_ continued. Approximately 85% of the quantity was removed in the first H_2_SO_4_ solution (1. Drag-out ${{\mathrm{NH}}_{4}}^{+}$). Nearly 15% in the second solution (2. Drag-out ${{\mathrm{NH}}_{4}}^{+}$) and less than 0.5% in the third one (3. Drag-out ${{\mathrm{NH}}_{4}}^{+}$). The average totaled amount of NH_3_ that was expelled from the membrane is displayed as the blue bar. It can be concluded that the quantity of ${{\mathrm{NH}}_{4}}^{+}$ that was previously exchanged into the membrane has been recovered within the margin of error.

### 4.2. Ammonia Desorption in the Electrochemical Cell

#### 4.2.1. Ammonia Desorption

**Figure 8:**
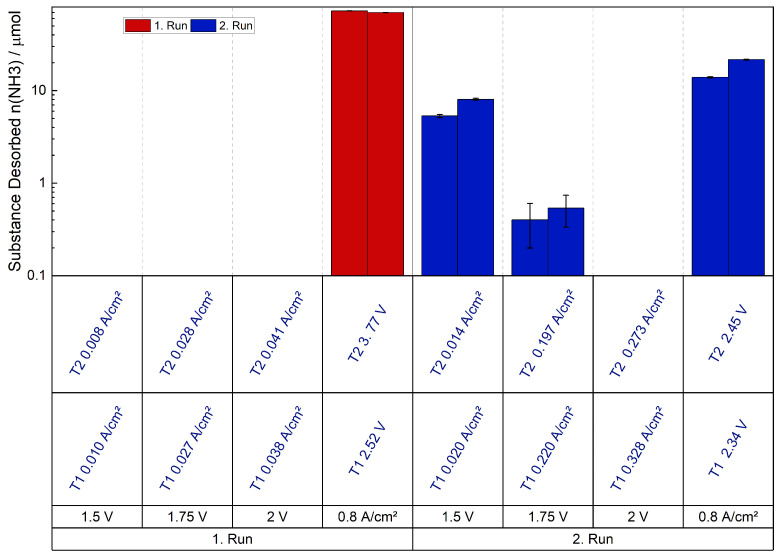
Expelled amount of NH_3_ of the 2:1 ratio, a logarithmic scale is used. The red color represents the measurement protocol of the first run and the blue of the second run. The applied potentials/current densities are written in black, while the measured potentials/current densities are written in blue.

Figure 8 displays the desorption of NH_3_ during the trials of a ratio of 2:1 NH_4_^+^ vs. H^+^ (c(NH_4_^+^) = 0.032 mol L^−1^) at ambient temperature. The measurement protocol was repeated with a fresh cell, including a fresh MEA, to ensure reproducibility. The first bar of each measurement point is attributed to the first trial (T1), and the second bar is attributed to the second trial (T2). The measurement protocol was carried out in its entirely twice, with each measurement point held for a duration of 30 min. At the beginning of each run, an impedance spectrum was recorded. The impedance spectra will be subsequently discussed. For the splitting of water and the generation of $H^{+}$, which are needed to drive ${{\mathrm{NH}}_{4}}^{+}$ out from the membrane, an electrical potential is required. For this purpose the potentials 1.5, 1.75, and 2 V were applied, which are common for water electrolysis [37]. As these potentials did not lead to the desorption of NH_3_, the maximum current supply of the potentiostat, 5 A, resulting in a current density of 0.8 A cm^−2^, was applied in order to expel NH_3_. After the current supply, a measurement run was finished and the next started with the stated potentials.

The values are plotted logarithmically and each run is illustrated in a different color. The graphical representation exclusively presents bars corresponding to conditions that lead to NH_3_ desorbed quantities surpassing the margin of the error of 0.2 μmol. During the applications of the current density a considerable quantity of NH_3_/${{\mathrm{NH}}_{4}}^{+}$ was expelled from the membrane in proportion to the quantity expelled while the potentials were applied. The desorption rate declines with the second run when the highest current density was applied. In the case of the second run, the determined desorption was only 25% of that determined in the first run. No desorbed NH_3_ was detected in the margin of error in the initial run when the potentials were applied and in the second run applying 2 V. However, NH_3_ was detected in the subsequent run after the current density was applied. This was unexpected as in the first run no NH_3_ was detected at 1.5 V. Therefore, it is assumed that the ${{\mathrm{NH}}_{4}}^{+}$-ions were no longer ionically bound in the membrane. They were released by the previous current supply, converted to NH_3_, remained in the cell and subsequently left the cell by diffusion. The current densities exhibited an increase from the first run to the second, relating to the applied potentials. This increase in current density was attributed to the driving out of NH_4_^+^ from the membrane, leading to an enhancement in ionic conductivity and thus an increase in the current density.

**Figure 9:**
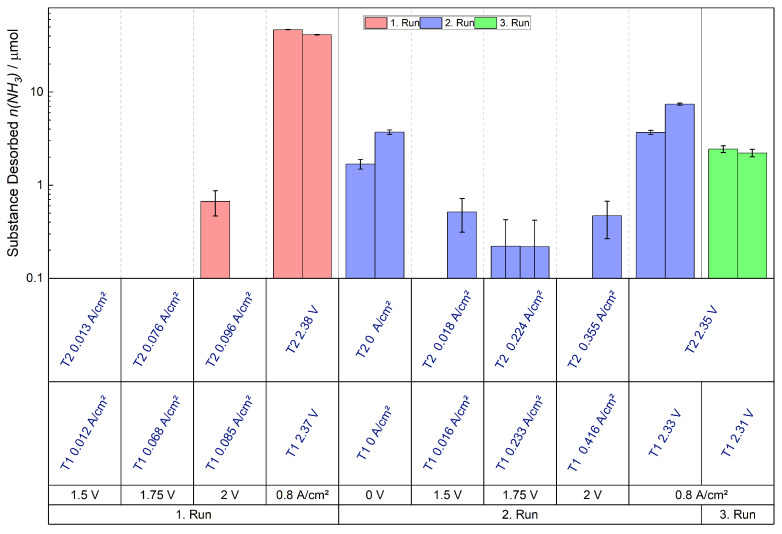
Expelled amount of NH_3_ of the 1:1 ratio, a logarithmic scale is used. The red color represents the measurement protocol of the first run, the blue the second, and the green the third run. The applied potentials/current densities are written in black, while the the measured potentials/current densities are written in blue.

A 1:1 ratio of NH_4_^+^ vs. H^+^ was employed (c(NH_4_^+^) = 0.016 mol L^−1^) to study when a different ratio has a major influence on the desorption behavior (Figure 9) at ambient temperature. The absolute desorption of NH_3_ was lower, yet the qualitative desorption was highly comparable and the experiment was also reproducible. Furthermore, the behavior subsequent to the initiation of the current density of 0.8 A cm^−2^ was investigated. Therefore, the potential was set to 0 V, precluding any further electrochemical reaction that results in an NH_3_ desorption. However, NH_3_ was driven out at the dead-voltage state, as depicted in Figure 9. The results underscore that the ${{\mathrm{NH}}_{4}}^{+}$-ions were no longer ionically bound, but already in the NH_3_ state and were subsequently removed by diffusion. Hence, the desorption of NH_3_ was not solely contingent upon electrochemical processes driven by potential, but diffusion also plays a contributory role. Apparently, some NH_3_ was removed from the membrane under the current flow and persisted in another part of the cell, from where it could be expelled by a purely diffusive process in the subsequent step with no potentials applied. Additionally, a third run was executed at 0.8 A cm^−2^. The trend continuous and the desorbed quantity declines with successive runs.

**Figure 10:**
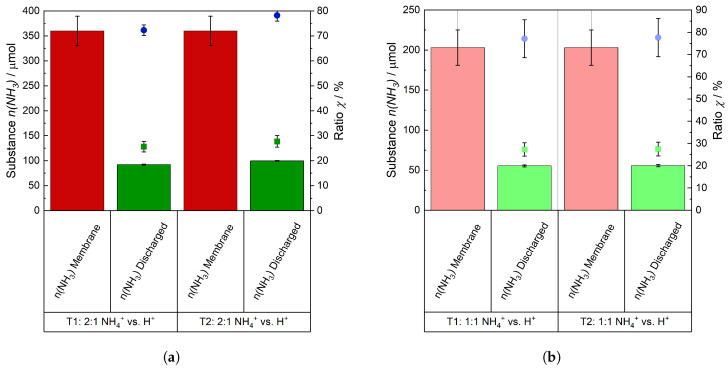
Comparison and ratio of the expelled amount of NH_3_ (green bars) compared to the initial amount of NH_3_ in the membrane (red bars). Green dots refer to the ratio of the expelled amount to the initial amount in the entire membrane while blue dots refer to the ratio of the expelled amount to the initial amount in the active area of the membrane only. (**a**) Ratio of 2:1 NH_4_^+^ vs. H^+^ and (**b**) ratio of 1:1 NH_4_^+^ vs. H^+^.

In order to demonstrate the transparency and reliability of NH_3_ desorption and the resulting consequences of this on the properties of the membrane, both trials of the respective ratios of 1:2 and 1:1 of ${{\mathrm{NH}}_{4}}^{+}$ vs. $H^{+}$ are shown in Figure 10.

The expelled quantity of NH_3_ was totaled in the respective experiments to determine whether the amount that was ion-exchanged into the membrane was expelled. This result is displayed in Figure 10. Figure 10a displays the outcome of the 2:1 ratio and Figure 10b the outcome of the 1:1 NH_4_^+^ vs. H^+^ ratio. The determined expelled quantities of NH_3_ are shown as green bars and in red are shown the calculated quantities of NH_3_/NH_4_^+^, which were in the membrane at the start of the trials. The red bar is based on the subtraction of the average value from the initial NH_4_^+^ concentration of the (NH_4_)_2_SO_4_ solution and the NH_4_^+^ concentration in the solution after the ion exchange. The ratio of the values (green bar divided by the red one) for each experiment is plotted as green square dots on the right-hand y-axis. It can be seen that only 25–30% of the NH_3_ present in the whole membranes were desorbed from them. It must be taken into account that the active cell areas of the membranes, where the electrochemical reactions occur, were 6.25 cm^2^, whereas their total areas were 17.64 cm^2^. The ratios of the desorbed quantities in relation to those in the active cell area are shown as blue dots. A significant proportion of the ${{\mathrm{NH}}_{4}}^{+}$-ions, between 70 and 80%, in the active cell area were expelled. The remaining 20 to 30% of the ${{\mathrm{NH}}_{4}}^{+}$-ions were still chemically bounded to the sulfonic acid groups within the membrane.

**Figure 11:**
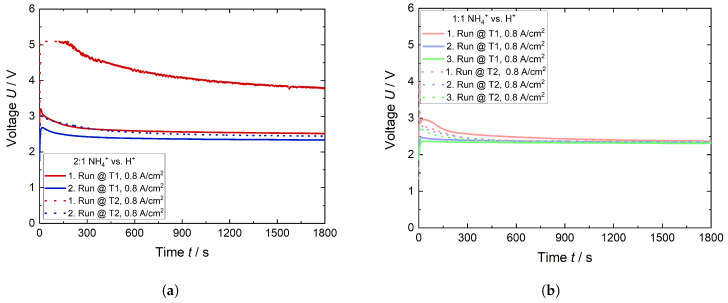
Galvanostatic experiments for both ratios 2:1 and 1:1 NH_4_^+^ vs. H^+^ at room temperature. (**a**) Ratio of 2:1 NH_4_^+^ vs. H^+^ and (**b**) ratio of 1:1 NH_4_^+^ vs. H^+^.

Galvanostatic experiments performed at the maximum current density of 0.8 A cm^−2^ are presented in Figure 11. On the left-hand side are shown the results of the 2:1 ratio and on the right-hand side those of the 1:1 NH_4_^+^ vs. H^+^ one. The measured voltages were highest during the first run, especially during the second trial of the 2:1 NH_4_^+^ vs. H^+^ (T2) ratio. Apart from this, the voltages decreased up to approximately 600–800 s. Afterwards, they remained constant over time. Compared to the first run, the potentials decreased in the second. This decrease was particularly pronounced in T2 of the 2:1 NH_4_^+^ vs. H^+^ ratio. It is assumed, that in the case of T2, the quantity of the ${{\mathrm{NH}}_{4}}^{+}$-ions within the active cell area was larger in comparison to T1. However, as a consequence of the elevated current supply during the initial run, approximately 57% of NH_3_, which was bounded as ${{\mathrm{NH}}_{4}}^{+}$ to the sulfonic acid groups in the channel-shaped microstructure of Nafion in the active cell area, was expelled from the membrane. This desorption resulted in the liberation of sufficient channels from ${{\mathrm{NH}}_{4}}^{+}$-ions, thereby drastically increasing the conductivity of $H^{+}$ through the membrane. As a result, a significant reduction in the potential for the second run of T2 was observed, so that the measured potential is similar to that from T1. No substantial change in measured potentials was evident from the second to the third run due to the fact that the remaining ${{\mathrm{NH}}_{4}}^{+}$-ions within the active cell area of the membrane no longer interact with the incoming $H^{+}$. Therefore, the membrane resistivity decreased, leading to a reduction in the required potential for the current flow.

**Figure 12:**
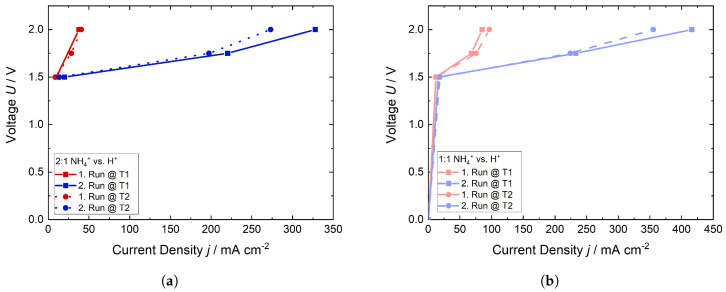
Polarization curves of the trials for both ratios 2:1 and 1:1 NH_4_^+^ vs. H^+^ at room temperature. (**a**) Ratio of 2:1 NH_4_^+^ vs. H^+^ and (**b**) ratio of 1:1 NH_4_^+^ vs. H^+^.

Figure 12 shows the respective polarization curves, to which potentials were applied and the current densities measured. During the first run, the measured current densities were low at the potentials of all trials. After the first time 0.8 A cm^−2^ was applied, a significant amount of NH_3_ was expelled, resulting in a substantial decrease in the membrane’s resistivity. Thus, the current densities, which were measured during the applied potentials, increased as shown in Figure 12. Notably, the current densities recorded for the 1:1 were found to be higher, which could be attributed to a lower total NH_4_^+^ content within the membrane, leading to enhanced conductivity.

**Figure 13:**
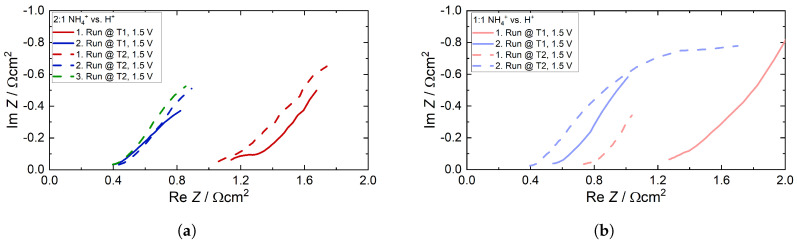
Impedance spectra of the trials and their runs at 1.5 V at room temperature. (**a**) Ratio of 2:1 NH_4_^+^ vs. H^+^ and (**b**) ratio of 1:1 NH_4_^+^ vs. H^+^.

**Figure 14:**
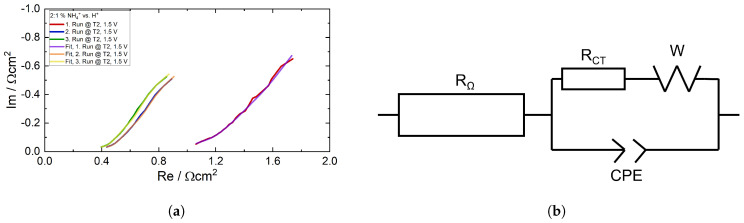
(**a**) Impedance spectra of T2 of the ratio 2:1 NH_4_^+^ vs. H^+^ with their corresponding fits at 1.5 V and (**b**) the Randles equivalent circuit used for fitting.

Figure 13 depicts the corresponding impedance spectra, which were recorded at the start of each run at the applied voltage of 1.5 V. Each spectrum consists of a high frequency (h.f.) semicircle and exhibits more or less linear behavior at low frequencies (l.f.) with a slope close to 45°. At the lowest frequencies down to 1 Hz, the impedance deviates from the linear slope, tending slightly towards the real axis. This suggests that the l.f. impedance is dominated by a finite diffusion process that can be modeled by, e.g., a finite Warburg impedance. The h.f. semicircle can be interpreted as the parallel connection of a kinetic resistance and a double layer capacitance. Together with the ohmic resistance, the overall impedance can be represented by the well-known Randles equivalent circuit, as shown in Figure 14b. Note that due to the depressed h.f. semicircle, the double layer capacitance was replaced by a constant phase element.

This replacement is most evident in the first run of T1 in Figure 13a, whereas the h.f. semicircle in the second run is not particularly distinct. In the case of T2, a third spectrum was recorded after the second run. A discernible reduction in the ohmic resistance from the first run to the second occurred in both ratios. The impedance spectra emphasize that the presence of NH_4_^+^ within the membrane initially has a considerable influence on the ohmic resistance, and therefore on the ion conductivity. However, once a certain quantity is expelled, the remaining NH_3_/NH_4_^+^ in the membrane no longer has a significant influence on the ohmic resistivity, as the ohmic resistance of the third run in Figure 13a is highly comparable to the ohmic resistance of the second run.

In order to investigate the electrochemical change in more detail, the spectra of T2 of the ratio 2:1 NH_4_^+^ vs. H^+^ were fitted (Figure 14a) by means of the Randles circuit shown in Figure 14b. The spectrum of the first run was fitted in a frequency range from 1000–1 Hz and those of the second and third ones were fitted from 1200– 1 Hz. The values of the electrical elements are shown in Table 2. The fits of the other trials and their corresponding tables can be found in the Supplementary Information. The ohmic resistance (*R*_Ω_), the charge transfer resistance (*R_CT_*), the diffusion resistance (*W_R_*), and the diffusion parameter (*W_T_*), have in common that they decrease by a factor of 2–3 from the first to the second runs. Conversely, with respect to the fitting error, these parameters remain virtually constant from the second to the third runs. The Warbung exponent (*W_P_*) also remains constant from the first to the third runs. It is close to the ideal value of 0.5 that corresponds to a 45° line in the Nyquist plot. The significant error of the constant phase element, which represents the double layer capacitance does not allow the analysis of both the CPE value (*CPE_T_*) and exponent (*CPE_P_*).

Although the significant decrease in the ohmic resistance from the first to the second run is clearly due to the exchange of NH_4_^+^ by H^+^, the corresponding decrease in the other parameters requires some explanation. *R_CT_* can be related to the oxygen evolution reaction (OER) at the anode, the hydrogen evolution reaction (HER), and the desorption of NH_3_ at the cathode. If the OER is assumed to be less affected by the cation exchange in the membrane compared to the cathode reaction, it might be hypothesized that the decrease in the charge transfer resistance from the first to the second run is caused by an increasingly dominating HER. The HER becomes dominating, because less NH_4_^+^ is present in the membrane, leading to a better H^+^ transport in it. The corresponding decrease in *W_R_* must be related to the acceleration of a diffusion process. Again, if the OER is negligible, the diffusion velocity of the H^+^ increases, because the NH_4_^+^ in the membrane, which slows down the H^+^ diffusion velocity, diminishes. *W_T_* relates to the effective diffusion length (*L*) and the diffusion coefficient (*D*), with the equation $W_{T}=\frac{L^{2}}{D}$. If the diffusion length is assumed to be approximately constant, the higher diffusion coefficient of protons compared to NH_4_^+^ could explain the decrease in *W_T_*. Alternatively, if *W_T_* is only related to the diffusion of H^+^ and considering Fick’s law (Equation (8)),

(8) $$J=D\;\frac{dC}{dx}$$

the diffusion coefficient is inversely proportional to the concentration gradient ($\frac{dC}{dx}$). This means that if the concentration of H^+^ increases within the membrane due to less NH_4_^+^ within the membrane and less resistivity, the diffusion coefficient decreases, as does the effective diffusion length. *J* is the flux of the diffusion species.

#### 4.2.2. Whereabouts of Ammonia

**Figure 15:**
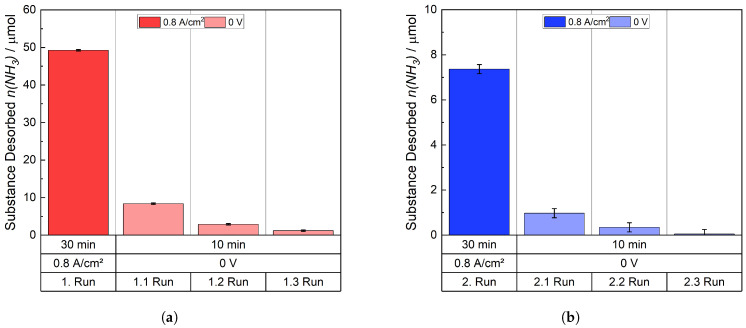
Expelled amount of NH_3_ during the applied current density and in the dead-voltage state. (**a**) Desorption 1. Run and (**b**) Desorption 2. Run.

To address the questions concerning the whereabouts of NH_3_/${{\mathrm{NH}}_{4}}^{+}$, the experiments were repeated, the cell was disassembled, and the individual MEA-components were also immersed in an H_2_SO_4_ solution so that the amount of NH_3_/${{\mathrm{NH}}_{4}}^{+}$ contained in them was expelled. Moreover, the time trend of the drag-out in the dead-voltage state was examined in greater detail. An MEA cell with Nafion 115, which was ion-exchanged with a solution of an NH_4_^+^ concentration of 0.024 M (1.5:1 NH_4_^+^ vs. H^+^), was utilized. The concentration was chosen to determine whether the desorption of NH_3_/${{\mathrm{NH}}_{4}}^{+}$ under the specific conditions was proportional to the ion exchange. Figure 15 displays the amount of NH_3_ detected during the runs. In the first one, 49.23 μmol was driven out at 0.8 A cm^−2^ and without an applied voltage 12.45 μmol. In the case of the second run, it was 7.37 μmol and 1.37 μmol, respectively. Table 3 shows the quantity and ratio of the expelled NH_3_ (Σn*_tot_*) during the dead-voltage state and the diffusion-driven drive-out, in comparison to the total drive-out. The corresponding drive-out during the time intervals (V1–V3) is also shown. Approximately 20–25% of the quantity of NH_3_, which desorbed while supplying 0.8 A cm^−2^, was expelled when no voltage was applied. Approximately two-thirds of this expelled quantity was driven out within the first ten minutes. In the second time interval, around 25% was removed, whereas 3–10% was removed in the last 10 min. The error of the second run increased strongly. Additionally, the ratios exhibited minor deviations in a second trial. This deviation along with the augmented error observed in the second run, can be attributed to the minimal absolute values of the measured NH_3_. Such low magnitudes are significantly more susceptible to inaccuracies when expressed in a percentage relationship.

**Figure 16:**
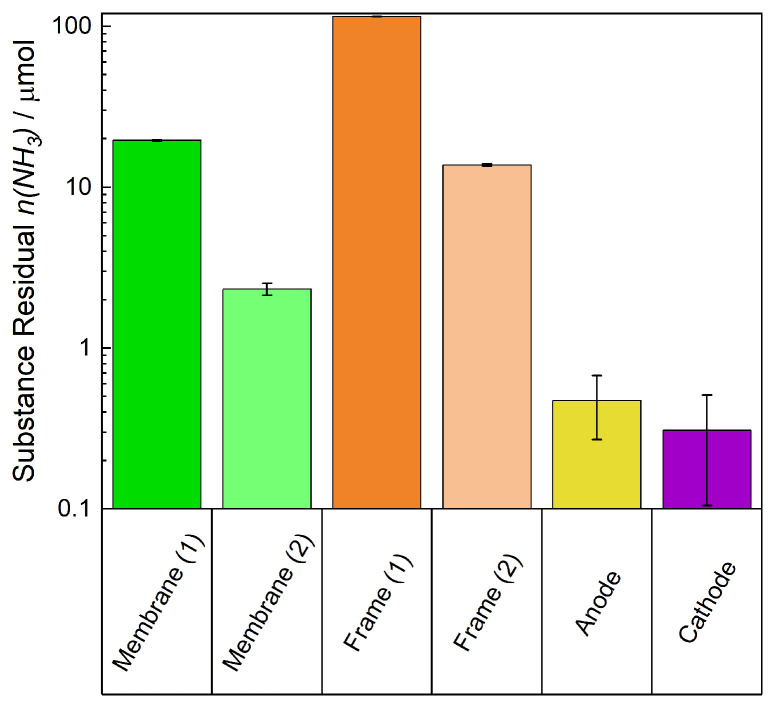
Detected amount of NH_4_^+^ remaining in the cell components, a logarithmic scale is chosen to show small amounts present in some components.

After the experiment had been completed, the cell was disassembled. The active cell area of the membrane was cut out and the active cell area, frame, anode, and cathode were immersed in H_2_SO_4_ solutions for 30 min. The portion of the active cell area and the frame that makes up the part of the membrane, which was covered by the gasket during the experiment, were re-inserted into a fresh solution because, as previously stated, not all of the NH_4_^+^-ions within the membrane were expelled in the first solution. The detected quantity of NH_3_ of each component is displayed logarithmically in Figure 16. Most of the detected quantity, 129 μmol, was found in the frame. This is based on the fact that no electrochemical reaction took place in the frame area but only in the active cell area. In the membrane itself, 22 μmol remained, which was detected. It is important to note that if the cell remains unused, exchange processes will take place so that NH_4_^+^ becomes homogeneously distributed within the membrane.

**Figure 17:**
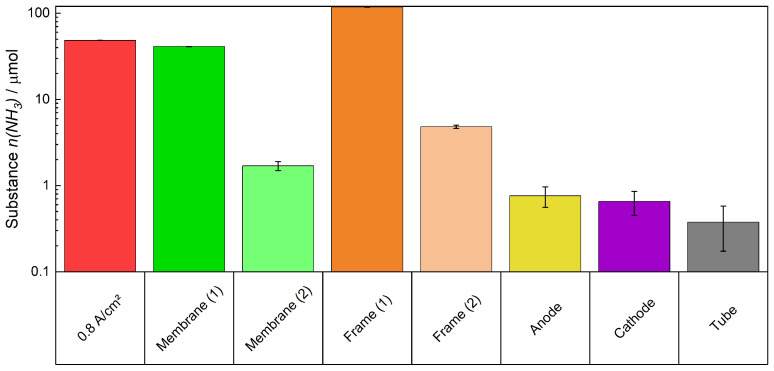
NH_3_ expelled electrochemical (red) and detected amount of NH_3_ expelled from the cell components. A logarithmic scale is chosen to show small amounts present in some components.

In the second trial, the cell was disassembled directly after applying a current density of 0.8 A cm^−2^. The NH_3_ desorption during the current flow is depicted in Figure 17. The desorption is similar to that in Figure 16, if it is assumed that there is a deviation like that in Figure 8 and Figure 9. Approximately 43 μmol of NH_3_ were expelled from the membrane after disassembling the cell and introducing the components in an H_2_SO_4_ solution. The quantity of NH_3_ is similar to the total amount of it expelled in the first test after applying 0.8 A cm^−2^ plus the remaining substance in the membrane. 122 μmol of NH_3_ were detected from the frame. The difference of 7 μmol compared to the amount depicted in Figure 16 is small when considering the deviation of the ion exchange in Figure 5. The determined amount of NH_3_ that was found in the electrodes and tube (the connection between the trap and cell) is low. Consequently, the residual amount of substance remained within the membrane. This result highlights the fact that the drag-out of ${{\mathrm{NH}}_{4}}^{+}$/NH_3_ from the membrane is an electrochemical and diffusion-driven process.

**Figure 18:**
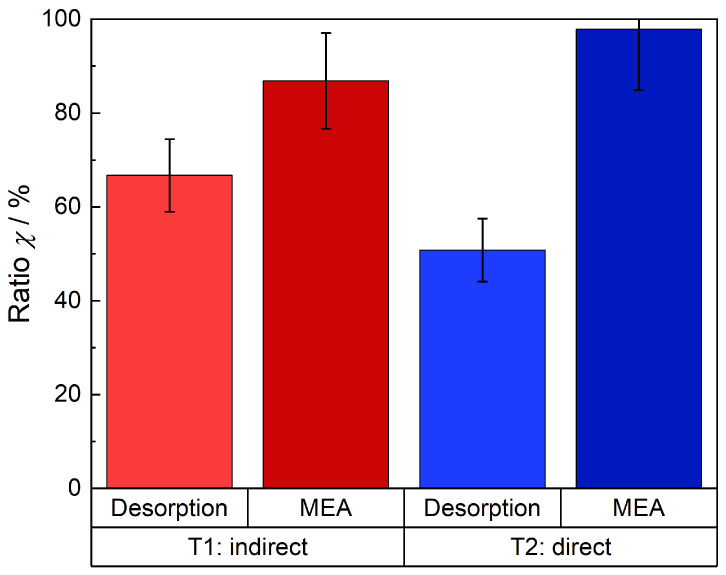
NH_3_ expelled electrochemical (red and blue bar) and the total amount of NH_3_ that was found, electrochemical plus the quantity which were expelled afterwards in the H_2_SO_4_ solutions from the cell components (dark red and blue).

Figure 18 displays the ratio of the detected quantities of NH_3_ in relation to the ion exchanged amount with respect to the active cell areas. T1 is the first trial where the time trend of the expelled NH_3_ was investigated (Figure 15 and Figure 16). The red bar illustrates a ratio of 67% ± 8%, denoting the amount of NH_3_ expelled electrochemically during the cell measurement in relation to the total ion exchanged amount. The quantity of NH_3_ released from the electrodes and the membrane when they were immersed into a H_2_SO_4_ solution after the electrochemical measurement is added to the electrochemical drag-out. Then a total quantity of 87% ± 10% of the NH_4_^+^-ions used previously within the membrane were expelled as NH_3_ from the membrane. In the case of the second trial where the cell was dismantled directly after applying the current, 51% ± 7% of the initial concentration of NH_4_^+^ in the membrane were expelled electrochemically (blue bar). This value is lower than during the first trial, because the quantity of NH_3_ that was expelled due to diffusion and when the high current density was applied for the second time, remained in the membrane. However, after disassembling and introducing the components in H_2_SO_4_ solutions, all of the introduced NH_4_^+^-ions in the beginning, were found (dark blue bar). In conclusion, it can be stated that the amount of NH_4_^+^ in the active cell area was almost completely recovered by the electrochemical discharge and chemical extraction.

## 5. Conclusions

In this study, we investigated the interaction of NH_3_ and Nafion, with the objective of enhancing understanding of the process, which will lead to more reliable ananlysis of electrochemical NH_3_ synthesis for future work. As expected, it was found that NH_4_^+^ prefers to exchange with H^+^ from Nafion, and the K_*eq*_ is 2.00 ± 1.31. This results in the persistence of ${{\mathrm{NH}}_{4}}^{+}$ within the membrane structure. The higher the concentration of the solution containing NH_4_^+^, the higher the exchange. It has been demonstrated that the quantity of ${{\mathrm{NH}}_{4}}^{+}$ bound to the membrane by adsorption is detected and expelled.

In the MEA cell, the effect of NH_4_^+^ in the membrane and also the migration of the ions in the different components was electrochemically investigated by analyzing the current-voltage behavior and impedance spectra, and chemically by analyzing the NH_3_/NH_4_^+^ content in the different components by dissembling the cell at different times in the electrochemical process utilizing the indophenol method. We found that the desorption of NH_3_ is a process driven by potential and diffusion. Considering the results and errors shown in Table 3, between approximately 10 and 30% of the amount of NH_3_ that was expelled electrochemically was done so by diffusion in the de-energized state. It was determined that a proportion (20 to 30%) of the protons in the active cell area can be replaced by ${{\mathrm{NH}}_{4}}^{+}$-ions, without affecting the ohmic resistance and ion conductivity. Consequently, if small amounts of NH_3_ are synthesized electrochemically in an N_2_ reduction cell, they may remain as ${{\mathrm{NH}}_{4}}^{+}$ in the membrane unnoticed as the membrane itself remains unaffected. Very high potentials > 2V are needed to expel a significant amount of NH_3_. However, theoretical calculations by Araujo et al. [38] indicate that NH_3_ synthesis is not feasible at these high potentials. It is shown in his article that these potentials lead to the complete coverage of the catalyst surface area with H*. For the N_2_* coverage, the potentials are small and the range is also very narrow. Therefore, it can be inferred that NH_3_ is detected under conditions in many cases that do not enable NH_3_ synthesis, leading to false conclusions. Additionally, it is shown that the majority of ${{\mathrm{NH}}_{4}}^{+}$ remains chemically bound in the membrane. Only a small proportion diffuses into the electrodes. Consequently, it is recommended to critically evaluate the obtained results, especially when detecting low amounts of NH_3_ and it is also necessary to examine the components of the synthesis system for the presence of NH_3_ before proceeding with further experiments. Otherwise, this is likely to result in subsequent experiments being influenced by the preceding one, thus affecting reproducibility and reliability.

## Figures and Tables

**Table 1:** Ion exchange concentrations.

NH_4_^+^ vs. H^+^	n(NH_4_^+^)	c(NH_4_^+^)	β(NH_4_^+^)	m((NH_4_)_2_SO_4_)
0.5:1	201.5 μmol	0.008 mol L^−1^	144 mg L^−1^	13 mg
0.8:1	322.4 μmol	0.013 mol L^−1^	235 mg L^−1^	22 mg
1:1	403 μmol	0.016 mol L^−1^	289 mg L^−1^	26 mg
1.5:1	604.5 μmol	0.024 mol L^−1^	433 mg L^−1^	40 mg
2:1	806 μmol	0.032 mol L^−1^	577 mg L^−1^	52 mg

**Table 2:** Elements of the impedance spectra of T2 of the ratio 2:1.

$R_{\Omega }$ [Ωcm^2^]	$R_{CT}$ [Ωcm^2^]	$W_{R}$ [Ωcm^2^]	$W_{T}$ [s]	$W_{P}$	${\mathrm{CPE}}_{T}$ [Ω^−1^ s^−1^ cm^−2^]	${\mathrm{CPE}}_{P}$
0.988±0.0250	0.231±0.0630	2.81±0.380	1.2±0.20	0.52±0.020	1.88±1.880	0.6±0.10
0.375±0.0310	0.119±0.0630	1.56±0.250	0.67±0.080	0.54±0.030	3.13±50	0.54±0.050
0.331±0.0380	0.119±0.0560	1.44±0.130	0.60±0.040	0.56±0.020	2.5±3.10	0.6±0.20

**Table 3:** Σn*_tot_* is the sum of the quantity of NH_3_, that was expelled during the runs in the dead-voltage states. V1 represents the first 10 time interval of 0 V, V2 the second, and V3 the third. χ is defined as the percentage of ${{\mathrm{NH}}_{4}}^{+}$ removed without applying a potential, relative to the amount removed at a current density of 0.8 A cm^−2^ for n*_tot_*. In addition, it represents the percentage of ${{\mathrm{NH}}_{4}}^{+}$ removed without applying a potential for the various time intervals, in relation to the total amount of ${{\mathrm{NH}}_{4}}^{+}$ removed without the application of a potential.

	1. Run		2. Run	
	n [μmol]	χ [%]	n [μmol]	χ [%]
Σ **n** *_tot_*	12.45±0.600	25.30±1.220	1.37±0.600	18.57±8.160
**V1**	8.38±0.200	67.31±3.620	0.98±0.200	71.15±34.460
**V2**	2.88±0.200	23.15±1.960	0.35±0.200	25.27±18.350
**V3**	1.19±0.200	9.54±1.670	0.05±0.200	3.57±15.700

## Data Availability

The data of this study are available from the corresponding author upon reasonable request.

## Supplementary Materials

The following supporting information can be downloaded at https://www.mdpi.com/article/10.3390/membranes15050149/s1, Figure S1: Temperature effect on desorption; Figure S2: Time effect on desorption; Figure S3: Impedance spectra and fits of T1, ratio 2:1; Table S1: Elements of the impedance spectra of T1 of the ratio 2:1; Figure S4: Impedance spectra and fits of T1, ratio 1:1; Table S2: Elements of the impedance spectra of T1 of the ratio 1:1; Figure S5: Impedance spectra and fits of T2, ratio 1:1; Table S3: Elements of the impedance spectra of T2 of the ratio 1:1.

## Abbreviations

The following abbreviations are used in this manuscript:

eNRR	Electrochemical nitrogen reduction reaction
MEA	Membrane electrode assembly
PTL	Porous transport layer
HER	Hydrogen evolution reaction

## References

[1] Xue Z., Zhang X., Qin J., Liu R. (2021). High-throughput identification of high activity and selectivity transition metal single-atom catalysts for nitrogen reduction. Nano Energy.

[2] Suryanto B.H.R., Du H.L., Wang D., Chen J., Simonov A.N., MacFarlane D.R. (2019). Challenges and prospects in the catalysis of electroreduction of nitrogen to ammonia. Nat. Catal..

[3] Schlögl R. (2003). Catalytic synthesis of ammonia—A “never-ending story”?. Angew. Chem..

[4] Tang C., Qiao S.Z. (2019). How to explore ambient electrocatalytic nitrogen reduction reliably and insightfully. Chem. Soc. Rev..

[5] Smil V. (1999). Detonator of the population explosion. Nature.

[6] Erisman J.W., Sutton M.A., Galloway J., Klimont Z., Winiwarter W. (2008). How a century of ammonia synthesis changed the world. Nat. Geosci..

[7] Hu L., Khaniya A., Wang J., Chen G., Kaden W.E., Feng X. (2018). Ambient Electrochemical Ammonia Synthesis with High Selectivity on Fe/Fe Oxide Catalyst. ACS Catal..

[8] Chanda D., Xing R., Xu T., Liu Q., Luo Y., Liu S., Tufa R.A., Dolla T.H., Montini T., Sun X. (2021). Electrochemical nitrogen reduction: Recent progress and prospects. Chem. Commun..

[9] Iriawan H., Andersen S.Z., Zhang X., Comer B.M., Barrio J., Chen P., Medford A.J., Stephens I.E.L., Chorkendorff I., Shao-Horn Y. (2021). Methods for nitrogen activation by reduction and oxidation. Nat. Rev. Methods Prim..

[10] van der Ham C.J.M., Koper M.T.M., Hetterscheid D.G.H. (2014). Challenges in reduction of dinitrogen by proton and electron transfer. Chem. Soc. Rev..

[11] Li K., Andersen S.Z., Statt M.J., Saccoccio M., Bukas V.J., Krempl K., Sažinas R., Pedersen J.B., Shadravan V., Zhou Y. (2021). Enhancement of lithium-mediated ammonia synthesis by addition of oxygen. Science.

[12] Shen H., Choi C., Masa J., Li X., Qiu J., Jung Y., Sun Z. (2021). Electrochemical ammonia synthesis: Mechanistic understanding and catalyst design. Chem.

[13] Wu T., Fan W., Zhang Y., Zhang F. (2021). Electrochemical synthesis of ammonia: Progress and challenges. Mater. Today Phys..

[14] Rafiqul I., Weber C., Lehmann B., Voss A. (2005). Energy efficiency improvements in ammonia production—Perspectives and uncertainties. Energy.

[15] Xu T., Liang J., Li S., Xu Z., Yue L., Li T., Luo Y., Liu Q., Shi X., Asiri A.M. (2021). Recent Advances in Nonprecious Metal Oxide Electrocatalysts and Photocatalysts for N 2 Reduction Reaction under Ambient Condition. Small Sci..

[16] Liu D., Chen M., Du X., Ai H., Lo K.H., Wang S., Chen S., Xing G., Wang X., Pan H. (2021). Development of Electrocatalysts for Efficient Nitrogen Reduction Reaction under Ambient Condition. Adv. Funct. Mater..

[17] Andersen S.Z., Čolić V., Yang S., Schwalbe J.A., Nielander A.C., McEnaney J.M., Enemark-Rasmussen K., Baker J.G., Singh A.R., Rohr B.A. (2019). A rigorous electrochemical ammonia synthesis protocol with quantitative isotope measurements. Nature.

[18] Kordali V., Kyriacou G., Lambrou C. (2000). Electrochemical synthesis of ammonia at atmospheric pressure and low temperature in a solid polymer electrolyte cell. Chem. Commun..

[19] Kumari S., Pishgar S., Schwarting M.E., Paxton W.F., Spurgeon J.M. (2018). Synergistic plasma-assisted electrochemical reduction of nitrogen to ammonia. Chem. Commun..

[20] Yang X., Nash J., Anibal J., Dunwell M., Kattel S., Stavitski E., Attenkofer K., Chen J.G., Yan Y., Xu B. (2018). Mechanistic Insights into Electrochemical Nitrogen Reduction Reaction on Vanadium Nitride Nanoparticles. J. Am. Chem. Soc..

[21] Lan R., Irvine J.T.S., Tao S. (2013). Synthesis of ammonia directly from air and water at ambient temperature and pressure. Sci. Rep..

[22] Leonardi M., Tranchida G., Corso R., Milazzo R.G., Lombardo S.A., Privitera S.M.S. (2022). Role of the Membrane Transport Mechanism in Electrochemical Nitrogen Reduction Experiments. Membranes.

[23] Liu H., Guijarro N., Luo J. (2021). The pitfalls in electrocatalytic nitrogen reduction for ammonia synthesis. J. Energy Chem..

[24] Hanifpour F., Sveinbjörnsson A., Canales C.P., Skúlason E., Flosadóttir H.D. (2020). Preparation of Nafion Membranes for Reproducible Ammonia Quantification in Nitrogen Reduction Reaction Experiments. Angew. Chem..

[25] Cai X., Iriawan H., Yang F., Luo L., Shen S., Shao-Horn Y., Zhang J. (2021). Interaction of Ammonia with Nafion and Electrolyte in Electrocatalytic Nitrogen Reduction Study. J. Phys. Chem. Lett..

[26] Hongsirikarn K., Goodwin J.G., Greenway S., Creager S. (2010). Influence of ammonia on the conductivity of Nafion membranes. J. Power Sources.

[27] Ren Y., Yu C., Tan X., Han X., Huang H., Huang H., Qiu J. (2019). Is It Appropriate to Use the Nafion Membrane in Electrocatalytic N 2 Reduction?. Small Methods.

[28] Yu T.H., Sha Y., Liu W.G., Merinov B.V., Shirvanian P., Goddard W.A. (2011). Mechanism for degradation of Nafion in PEM fuel cells from quantum mechanics calculations. J. Am. Chem. Soc..

[29] Ito H., Maeda T., Nakano A., Takenaka H. (2011). Properties of Nafion membranes under PEM water electrolysis conditions. Int. J. Hydrogen Energy.

[30] Jalani N.H., Datta R. (2005). The effect of equivalent weight, temperature, cationic forms, sorbates, and nanoinorganic additives on the sorption behavior of Nafion^®^. J. Membr. Sci..

[31] Zhao Y., Wu F., Miao Y., Zhou C., Xu N., Shi R., Wu L.Z., Tang J., Zhang T. (2021). Revealing Ammonia Quantification Minefield in Photo/Electrocatalysis. Angew. Chem..

[32] Li L., Tang C., Xia B., Jin H., Zheng Y., Qiao S.Z. (2019). Two-Dimensional Mosaic Bismuth Nanosheets for Highly Selective Ambient Electrocatalytic Nitrogen Reduction. ACS Catal..

[33] Shiva Kumar S., Himabindu V. (2019). Hydrogen production by PEM water electrolysis—A review. Mater. Sci. Energy Technol..

[34] Litster S., McLean G. (2004). PEM fuel cell electrodes. J. Power Sources.

[35] Halseid R., Vie P.J.S., Tunold R. (2004). Influence of Ammonium on Conductivity and Water Content of Nafion 117 Membranes.

[36] Morris D.R., Sun X. (1993). Water–sorption and transport properties of Nafion 117 H. J. Appl. Polym. Sci..

[37] Carmo M., Fritz D.L., Mergel J., Stolten D. (2013). A comprehensive review on PEM water electrolysis. Int. J. Hydrogen Energy.

[38] Araujo R.B., Edvinsson T. (2024). Supervised AI and Deep Neural Networks to Evaluate High-Entropy Alloys as Reduction Catalysts in Aqueous Environments. ACS Catal..

