# Supplementary material for: The Ammonia Adsorption and Desorption Behavior of Nafion
Source: Membranes (Basel). 2025 May 14;15(5):149. doi: 10.3390/membranes15050149 (PMC12112899; doi:10.3390/membranes15050149)
Supplement: Supplementary file 1 [file membranes-15-00149-s001.zip › membranes-3537902-supplementary.pdf]

Supplementary Materials: The Ammonia Adsorption and Desorption Behavior of Nafion

Dominik Sachse [1,3]*, Andreas Glüsen [1], Klaus Wippermann [1], Martin Müller [1], Uwe Rau [2,3] and Ralf Peters [1,4]

1. Supplementary Information

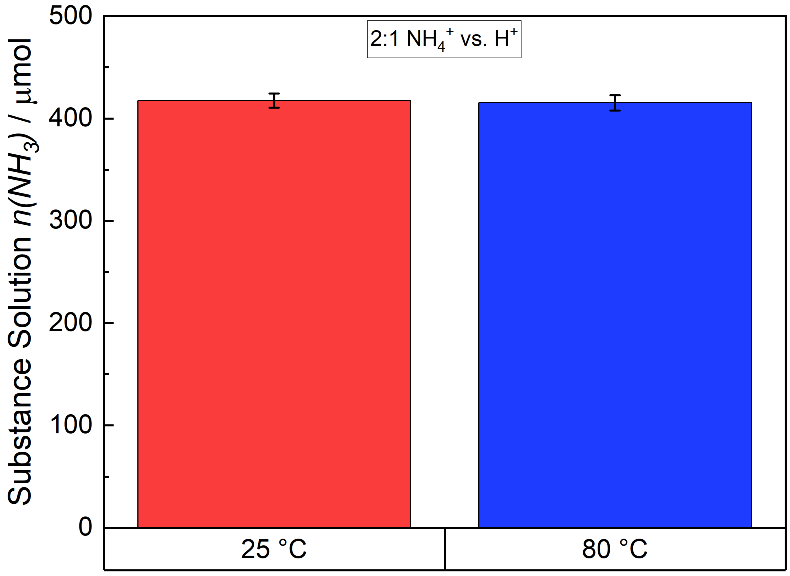

Figure S1. Desorption of NH_3 from the electrochemical cell executed at 25 °C (red bar) and 80 °C (blue bar). The ratio of NH_4^+ to H^+ is 2:1.

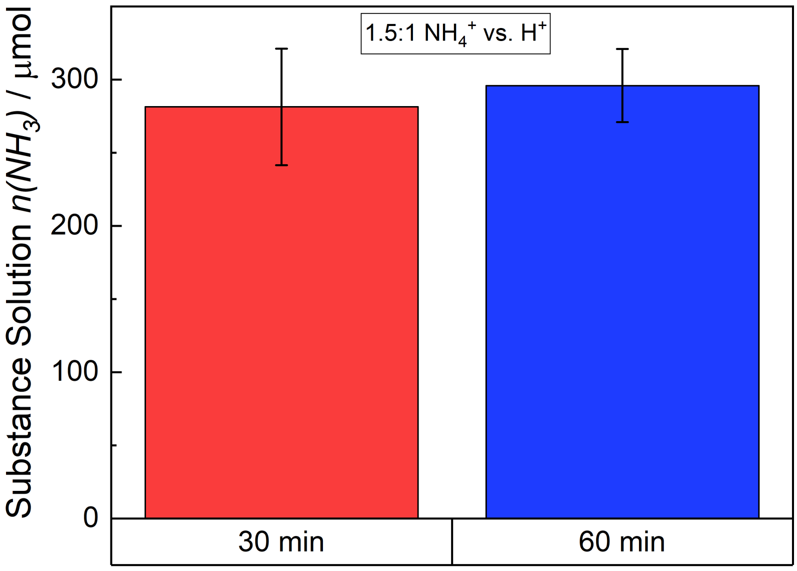

Figure S2. Desorption of NH from the electrochemical at 25 °C while varying the exchange time 30 min (red bar) and 60 min (blue bar). The ratio of NH_4^+ to H^+ is 1.5:1.

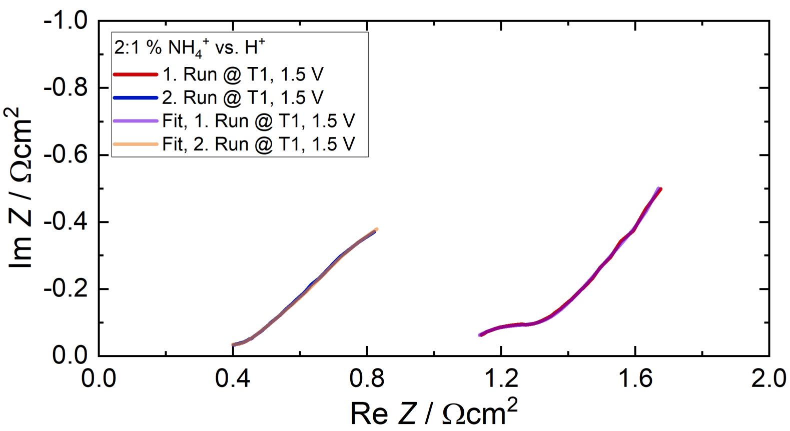

Figure S3. EIS of T1, 2:1 ratio (red and blue), and their respective fits (purple and orange).

Table S1. Elements of the impedance spectra of T1 of the ratio 2:1

R_{Ω} [Ω]	R_{CT} [Ω]	W_R [Ω]	W_T [F]	W_P	CPE_T [Ω^{-1} s^{-1}]	CPE_P
0.172 ± 0.001	0.044 ± 0.002	0.35 ± 0.01	1.28 ± 0.05	0.580 ± 0.006	0.17 ± 0.03	0.67 ± 0.02
0.049 ± 0.009	0.02 ± 0.01	0.197 ± 0.003	0.93 ± 0.04	0.469 ± 0.003	0.10 ± 0.07	0.6 ± 0.2

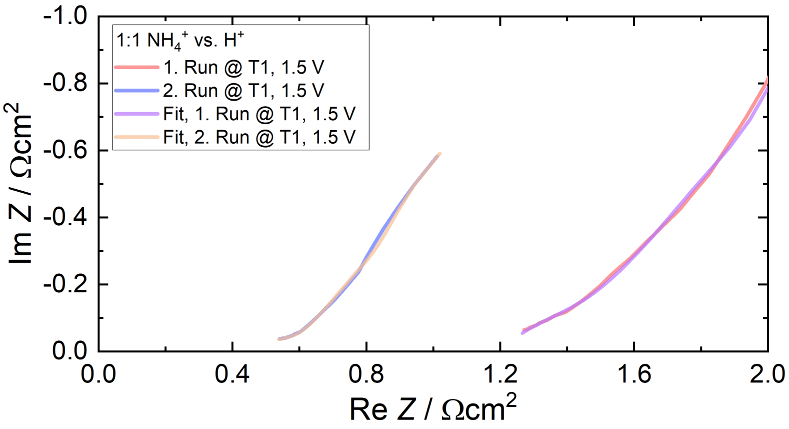

Figure S4. EIS of T1, 1:1 ratio (red and blue), and their respective fits (purple and orange).

Table S2. Elements of the impedance spectra of T1 of the ratio 1:1

R_Ω [Ω]	R_{CT} [Ω]	W_R [Ω]	W_T [F]	W_P	CPE_T [Ω^{-1} s^{-1}]	CPE_P
0.192 ± 0.002	0.08 ± 0.03	0.8 ± 0.2	0.9 ± 0.2	0.61 ± 0.04	0.7 ± 0.4	0.51 ± 0.07
0.076 ± 0.004	0.023 ± 0.008	0.29 ± 0.03	0.70 ± 0.06	0.57 ± 0.02	0.412 ± 0.05	0.6 ± 0.2

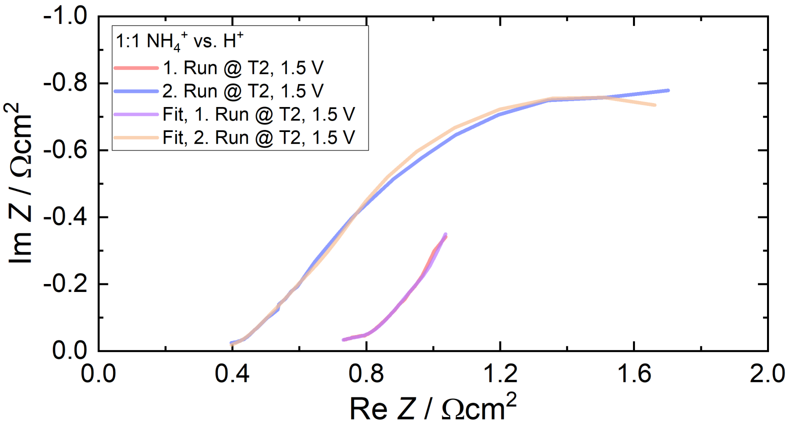

Figure S5. EIS of T2, 1:1 ratio (red and blue), and their respective fits (purple and orange).

Table S3. Elements of the impedance spectra of T2 of the ratio 1:1

R_Ω [Ω]	R_{CT} [Ω]	W_R [Ω]	W_T [F]	W_P	CPE_T [Ω^{-1} s^{-1}]	CPE_P
0.108 ± 0.002	0.027 ± 0.005	0.22 ± 0.02	0.94 ± 0.06	0.59 ± 0.02	0.8 ± 0.4	0.51 ± 0.08
0.059 ± 0.001	0.014 ± 0.002	0.34 ± 0.03	2.1 ± 0.1	0.54 ± 0.01	1.5 ± 0.4	0.51 ± 0.05
